# Network analysis: An indispensable tool for curricula design. A real case-study of the degree on mathematics at the URJC in Spain

**DOI:** 10.1371/journal.pone.0248208

**Published:** 2021-03-11

**Authors:** Clara Simon de Blas, Daniel Gomez Gonzalez, Regino Criado Herrero

**Affiliations:** 1 Area de Estadistica e Investigacion Operativa, ETSII, URJC, Mostoles, Spain; 2 Instituto Universitario de Evaluacion Sanitaria, UCM, Madrid, Spain; 3 Departamento de Estadistica y Ciencia de los Datos, Facultad de Estudios Estadisticos, UCM, Madrid, Spain; 4 Departamento de Matematica Aplicada, Ciencia e Ingenieria de los Materiales y Tecnologia Electronica, ESCET, URJC, Mostoles, Madrid, Spain; 5 Center for Computational Simulation, UPM, Pozuelo de Alarcón, Spain; 6 Data, Complex Networks and Cybersecurity Research Institute, URJC, Madrid, Spain; Brigham Young University, UNITED STATES

## Abstract

Content addition to courses and its subsequent correct sequencing in a study plan or curricula design context determine the success (and, in some cases, the failure) of such study plan in the acquisition of knowledge by students. In this work, we propose a decision model to guide curricular design committees in the tasks of course selection and sequencing in higher education contexts using a novel methodology based on network analysis. In this work, the local and global properties stemming from complex network analysis tools are studied in detail to facilitate the design of the study plan and to ensure its coherence by detecting the communities within a graph, and the local and global centrality of the courses and their dependencies are analyzed, as well as the overlapping subgroups and the functions and different positions among them. The proposed methodology is applied to the study of a real case at the Universidad Rey Juan Carlos.

## Introduction

Many real world systems are subject to be modeled by the use of networks. In these networks nodes represent the different elements of the system and edges stand for the interactions or relationships between them [1]. In network analysis, a key point is to devise measures suitable for quantifying the strategic importance of a node, an edge, a set of nodes, or a set of edges, with the goal of identifying the optimal functioning of the system represented by the network [2–5]. Topological measures, such as different versions of vulnerability, efficiency, centrality, and clustering, can be used to quantify, compare, and rank different configurations for a specific system [2, 6–13]. A task specially difficult to solve for the university management authorities is the design of the different curriculum plans. Few general methodologies have been reported in the literature. Most of them are given in educational laws in general frameworks.

The TUNING Educational Structures [14] is a European project aimed at implementing the political objectives of the Bologna Process and the later Lisbon Strategy in the higher educational sector. This is an approach to redesigning, developing, implementing, evaluating, and enhancing the quality of undergraduate and graduate degree programs. The tools that can be found in TUNING Educational Structures are described in a range of publications which institutions and researchers are invited to test and use in their own settings as points of reference, convergence, and common understanding. The use of techniques derived from Network Analysis (NA) have been very useful in similar studies in which it is desired to know the strengths and weaknesses as well as the inconsistencies of an organization system (see for example some Management cases in [15–17]).

The majority of the studies that combine the use of networks in the design of curricula have been carried out through dependency graphs. Dependency graphs have generally been applied to situations where edges represent a temporary constraint in the planning of a global activity ([18–21]). For example, in a dependency graph, the links (A, B); (B, C) simultaneously implies that the activity A (course A) must be carried out before B. So, B cannot be carried out before activity A has been completely finished. In dependency graphs, transitivity is always imposed in this way, so there is no need to represent the link (A,C) since it is obviously deduced as a natural consequence of transitivity that any dependency graph must have. So, this dependency graphs are usually modeled as Directed Acyclic Graphs (i.e. DAG networks).

In this scenario, the main aim is to establish a temporal planning in order to establish the critical activities (critical activities are considered to be those ones producing a global delay in the global process). Another class of networking approach to curriculum study that is worth mentioning is based on how students move from one course to another throughout their careers [22]. In this way, it is possible to build a network that shows the real temporal relationships between courses. Combining historical data of the students and courses when available with networking techniques, improves students and university managers capacity to estimate and predict the courses in which a student will enroll in the future. This allows to have a forecast of the enrollment in the courses for next year. Interesting network analysis or this type of curricular graphs can be found in which central courses are determined by means of centrality measures ([22–24]). The main difference with other network modelling is that the relationships between courses are based on temporality and they are not known a priori. In other words, there is a link (i, j) between the courses i and j if many students have taken the course j right after having completed course i.

In this paper, we propose an influence network model for the curriculum plan design in which the links among courses reflect the influence that have some subjects over others due to temporal sequencing of content acquisition. The curriculum model proposed here responds to a system of recommendations which is not necessarily restrictive, allowing the student to take several courses simultaneously ([25–27]). It is easy to see that the influence/recommendation graph analyzed here shows a more general scenario than a system of prerequisites. So all of the analysis we have done in this work are subject to be carried out in a dependency graph found in the literature in which the transitivity is imposed and the arcs are not valued. The network of this work is built based on the course contents that are “necessary/recommended” for other courses. The objective of using network analysis for this recommendation/influence structure is to show (in addition with any classical temporally analysis) the robustness, centralization, cohesion and in general to understand the whole plan structure to be able to modify it if appropriate or necessary. The information obtained after an analysis of the network of influence (as proposed in this document) enriches the knowledge of the training itinerary.

The main aim of the research is to present a tool for a better understanding of the complex structure of a curriculum plan. It could be useful to construct a degree training itinerary as an interactive process that permits (by means of a network visualization, some network measures and analysis) assign scheduled/allocated courses to semesters, modified courses contents in case of inconsistencies and to understand in general the whole flow knowledge process. This article is organized as follows. In Section 2, we describe some preliminary concepts in network analysis. In Section 3, we describe our methodology for creating a network representing the relationships between courses in an undergraduate curriculum, as well as the networking measures considered used here for evaluating and interpreting the network’s properties. In Section 4 we describe the process of developing a degree’s curriculum in Spain. In Section 5, we present the results of applying the methodology in the design of the University Rey Juan Carlos (URJC) mathematics degree curriculum. And finally, in Section 6 the conclusions derived from this work are shown.

## Preliminaries

### Network analysis

#### Centrality measures

One of the most important problems in network analysis is the identification of key nodes and the relationships between nodes. In order to rank the nodes of a network, there exist many approaches, depending on the definition of “relevance” or “importance” as determined by network analysts. A common approach for ranking nodes in a network is to use centrality measures [12]. The considered centrality measure reflects the node’s (relative) importance inside the network. Although there are many centrality papers in literature [28, 29], centrality concept or idea is a complex notion that requires a clear definition. As it is pointed out in [3, 29], the use of a specific centrality measure implies some assumptions about the network structure and how information flows along the network, so it is very important to analyze first the class of network that you are modelling and after that use the adequate centrality measure. For example, in the shortest path (closeness or betweenness centrality measures), we only take into account the geodesic paths in the communication between each pair of nodes. Thus, it is assumed that information flows through the network only along the shortest feasible paths.

In this sense, it is important to mention that in this paper, we focus on centrality measures for dominance (or reference) networks where relationships between nodes are weighted and directed through a relation of precedence. For this type of networks, one of the most popular and most frequently used centrality measures is based on the degree of the node in the graph: the more edges incident at a node, the higher the node’s position in the ranking. Generally, however, nodes more central to the structure, in the sense of having higher degree or more connections, tend to be favored in the ranking. The degree in case of directed network can be obtained as the sum of the in-degree and out degree. In Program Evaluation and Review Techniques (PERT) [30] (which can be viewed as a class of dominance networks) the out-degree measure of the nodes can be used to classify the objects or activities in the project as leading or intermediate.

In network analysis the term “influence network” frequently appears to refer to directed networks in which the relations represent some degree of influence between nodes. For example, author citation networks or Twitter networks are often considered as influence networks. In those networks, if author j cites (or retweets) author i many times, we could say that author i has some influence over author j. In this sense, there is a significant difference with dependency or permission networks since in these situations the direct relation implies a stronger constraint, as for example, the transitivity between nodes. For example, in a permission network author j needs the approval of author i so this relation is something more than just an influence, or in a dependency network a node usually represents the instant in which a task is finished and the link between two nodes is associated with a task. So as a consequence, the relations should be transitive. Other term that also appears in many network analyses is the Recommendation networks. These are clearly closer to the concept of influence than dependency / permission networks. Recommendation networks are used to model friends (or similar users) recommendations about the order in which tasks have to be performed. For example, after reading the Book X, the system recommends (since your friends or similar users have also read this Book X) to read Book Y. These recommendations can be modelled as a directed or undirect network where the nodes are the books and the links are recommendations. From a mathematical point of view, dependency networks impose more conditions than recommendation or influence networks. Dependency networks are usually modelled as DAG.

Other measure that can be used for directed network is PageRank defined by Larry Page and Sergey Brin in [31], where the rank value indicates the importance of the node in the net.

Finally, other class of measures that deals with weighted and directed networks are the flow centrality measures [3, 32] that are based on regarding the network as representing a flow. In the case of the flow betweenness centrality measure, the contribution of a node represents the amount of flow that necessarily pass through this node in all possible maximum flows [32].

#### Community detection problems: Clustering nodes in a network

In addition to the centrality or importance of the nodes in a network, another topic that merits attention is the identification of groups or communities in a network. The identification of groups in a network is very useful for understanding the structure of the problem that we are analyzing. A network is called modular (see [33]) when its nodes are joined together in tightly knit groups among which there are only looser connections (i.e. if the nodes are naturally grouped into dense communities with few connections between communities). There are many algorithms that try to find communities in a network (see [34]). Such problems are commonly known as community detection problems.

Nevertheless, as with the topic of centrality measures, the use of a specific community detection method is not straightforward and is still an open problem the suitability of the proposed algorithms in the literature depending on the type of network that it is analyzed. The community detection problems for directed networks have been much less studied than the general ones, since the concept of group or community for directed networks is not clear as for non-directed networks [35].

**Fig 1:**
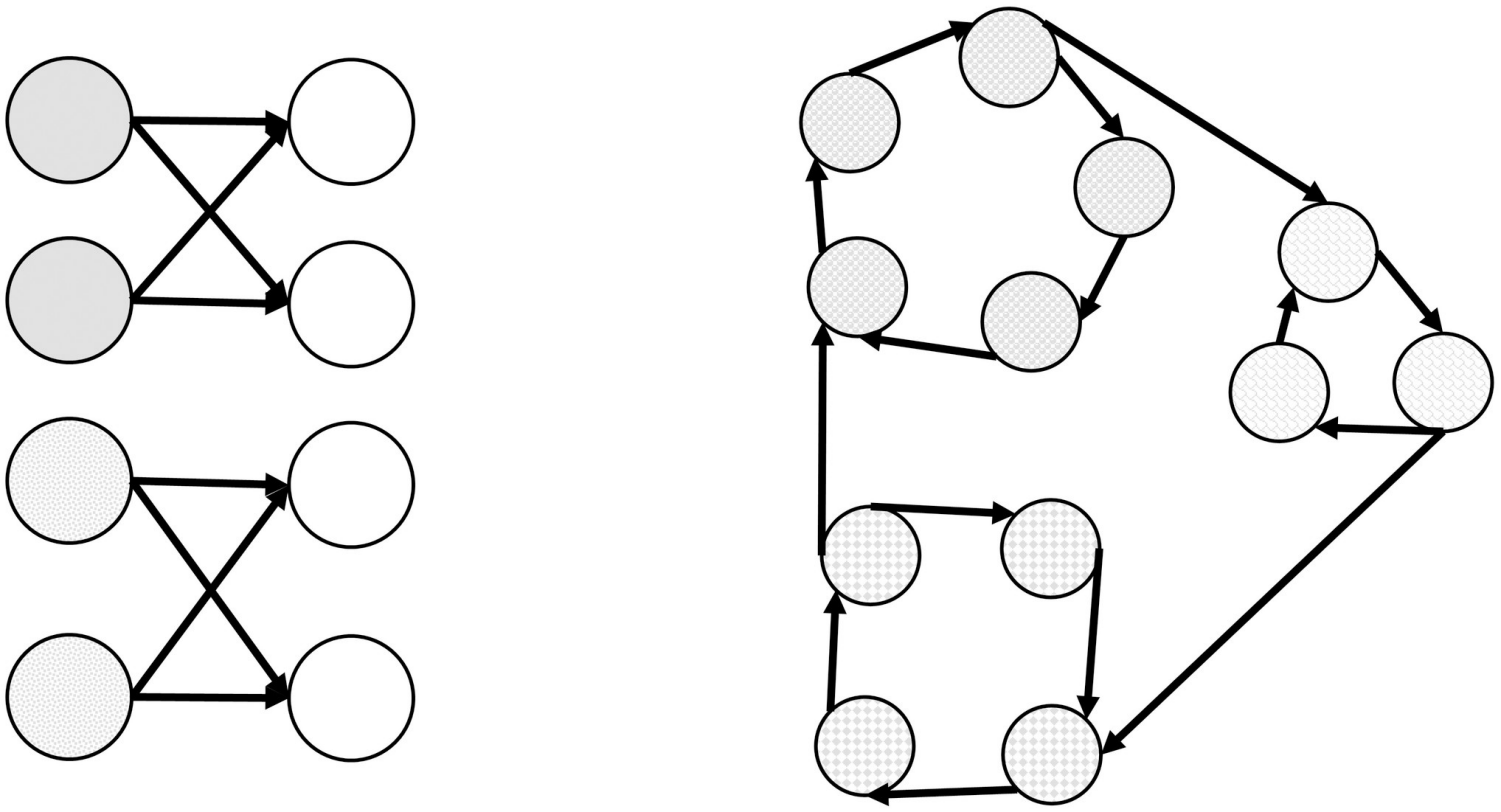
Citation-based cluster and flow-based cluster.

As pointed out in [35], the concept of group/community could be extended in different ways when changing from undirected networks to directed networks. In undirected networks the accepted interpretation of community/group is a set of members with many relationships between them and few relationships of them with other members outside the community. This community idea is related to the notion of density. Communities are dense subgraphs with lower relations with outside. In directed networks, this view is not the only way to consider communities. For example, if we have a directed network (on Twitter it is possible to follow someone who does not follow you), a group can consist of a set of nodes with similar “parents” even if they are not connected. For example, if two persons (non connected directly) who follow the same persons (politician, sport teams, university,..) we could say that they share characteristics in common and could be in the same community. This new community concept can be viewed in Fig 1 as citation-based cluster. Another notion of groups not necessarily similar to density is the idea of flow-based cluster, in which a group is a set of nodes that can communicated between them. Finally the idea of density (in the classical sense) is also adopted for the directed case. In this work, we have focused in density clusters.

Different algorithms [35] have been proposed for directed graphs depending on the class of clusters that you are looking for. The most common and simple approach (addressed as *clustering based on a naive graph transformation*) is to ignore edge directionality and treat graph as undirected. This methodology cannot capture citation-based clusters since the clusters could be very small or even disconnected (Fig 1). In other approaches (see [35]) the directed graph is converted into an undirected one, where the edge direction is meaningfully maintained in the produced network. There exist other methodologies based on modularity optimization algorithms that can be used to deal with directed networks in order to capture other class of community concepts.

#### Global network measures

Centrality measures are computed for each node of the network capturing the idea of central or importance. In this sense, centrality can be viewed as a local measure that does not give a general idea of how the network is structured. An aggregation of the different centrality measures (for example by means of the Gini Index or a variance of the centrality vector) could give us an idea of how the power in the network is distributed. This concept can be considered as a global property since makes reference of a quality of the whole network.

Another global network measure is the optimal modularity of a network (i.e. the best partition of the network in terms of the modularity measure) which permits us to know if a network presents groups or communities well-defined. If the modularity of this best partition is high we usually say that the network is modular.

Another relevant global measure is homophily. Homophily is an important concept aimed at encapsulating why the nodes in the network are linked. The general hypothesis is that nodes with similar characteristics are more likely to be connected. When this characteristic is the importance of the node (measured as the degree of incidence), it must be tested whether nodes with greater power are more likely to be connected among themselves than they are to be connected with lower power nodes. A coefficient measuring the correlation between the degrees of linked nodes can be used to capture this tendency.

Newman [4] introduces the assortativity measure, which makes it possible to classify networks into assortative and disassortive. A network is assortative if the correlation coefficient between the degree of nodes in the arc set is positive and significant nodes having many relations are connected amongst themselves with greater probability than they are connected with low degree nodes. In the opposite case of negative correlation coefficient, nodes with many connections tend to be connected with nodes with low total degree relations. However, some issues related to networks with different topologies presenting the same assortativity index or vice versa have been addressed in [36] who introduce, for undirected and unweighted networks, higher order assortativity based on a suitable choice of the matrix driving the connections. In this work, we have used the assortativity measure proposed by Newman [5] as a preliminary inspection to discover the network topology.

Taking into account that the network has been identified as assortative, it seems reasonable to find the existence of a core or a “rich club” in the network. A rich club is a set of very powerful nodes densely connected between them. This density reflects the tendency of hubs to be well connected with other hubs. This phenomenon can be described by the rich-club coefficient, introduced in [37]. The k-grade rich-club of a G network is defined to be the set of vertices R(k) whose degree is greater than k, and the k-grade rich-club coefficient is defined by

$$\begin{array}{c} \Phi \left(k\right)=\frac{2}{\left|R(k)|(|R(k)|-1\right)}\sum_{i,j\in R(k)}a_{ij}. \end{array}$$ (1)

To conclude this section, we will mention the motifs used to evaluate important substructures in the context of directed graphs. To search for repeated subgraphs having some well-defined structure, Davis, and Leinhardt [38] define a motif as a small connected subgraph having a particular given structure. It is argued that the motif profile (i.e. the number of different motifs in the graph) is characteristic for different types of networks and that network function is related to the motifs identified in the graph.

## Methodology: Using network analysis for curriculum design

As we have mentioned in the introduction, networks analysis has been used for the understanding of complex structures in order to analyze their strengths as well as their weaknesses. With the main objective of understanding the complex structure associated with a curriculum plan, we propose the following steps:

1. First step (Experimental design): build the network of influences between courses.
2. Second step (Network analysis): we propose some classical network analysis procedures to exploit the advantages of using network analysis tools and different applications that can be derived from the proposed measures, such as:
  - Inconsistencies identification.
  - Courses scheduling allocation in semesters.
  - Detection of key courses.
  - Detection of central courses.
  - Courses communities.
3. Third step (Results and conclusions): evaluate the network analysis measures in the curriculum plan.
4. Fourth step (iterative): present the information and the associated conclusions to the experts/professors for a better understanding of the complex structure and if it is necessary (due to some inconsistencies or unfeasibility in the semesters allocations) change the content of some courses to make reduce these inconsistencies and return to step 1.

**Fig 2:**
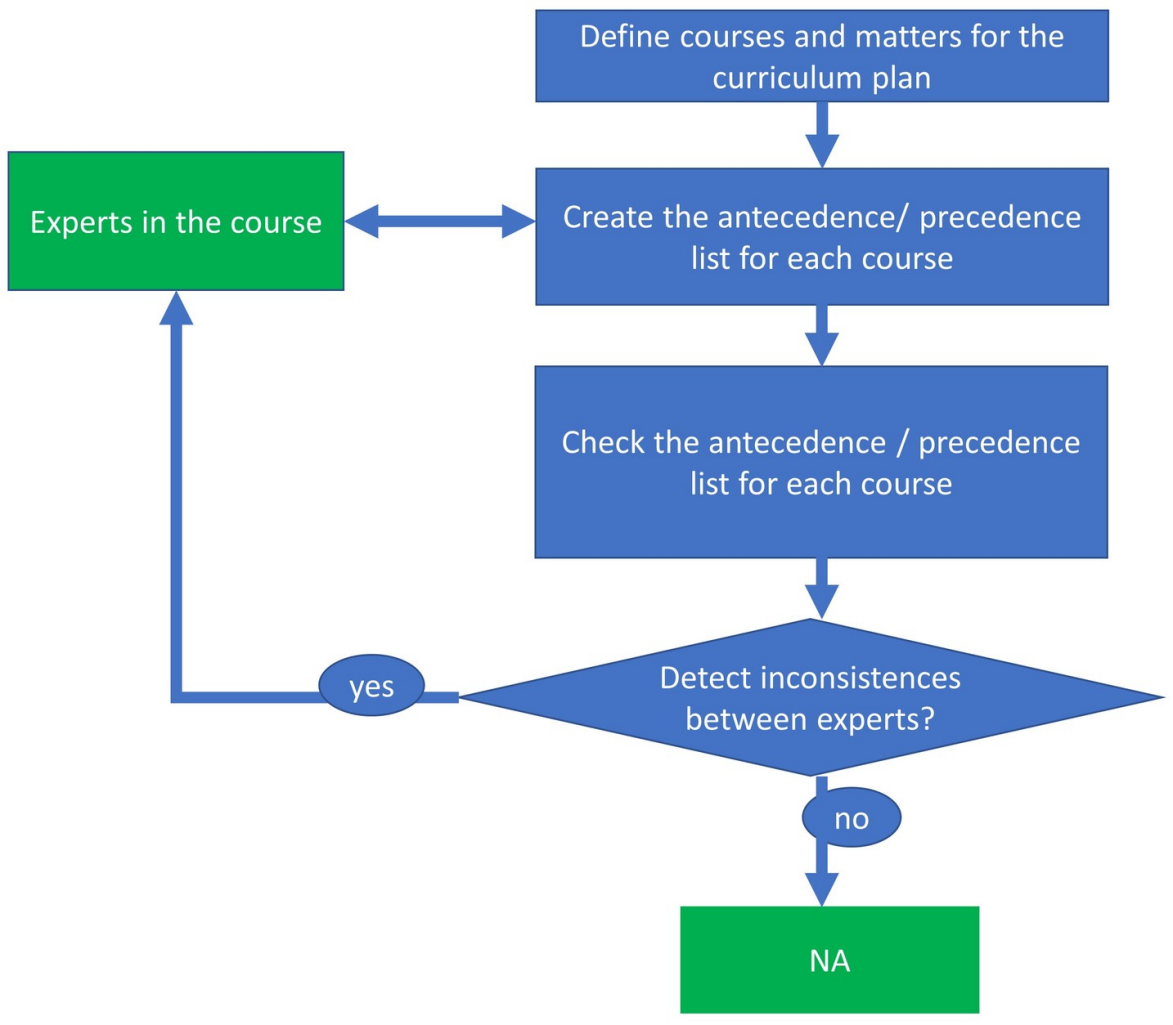
Decision aid model for the curriculum plan construction.

Fig 2 represents a decision aid model to construct the curriculum network.

In the first step we build the “Curriculum Network” that represents the relations between courses (based on the expert knowledge of the professors). For each course “A”, we have interviewed professors with more than 5 years of experience teaching lecture “A”. The professors establish the percentage of material in course B necessary to understand the contents of course A appropriately in a selected number of levels.

**Fig 3:**
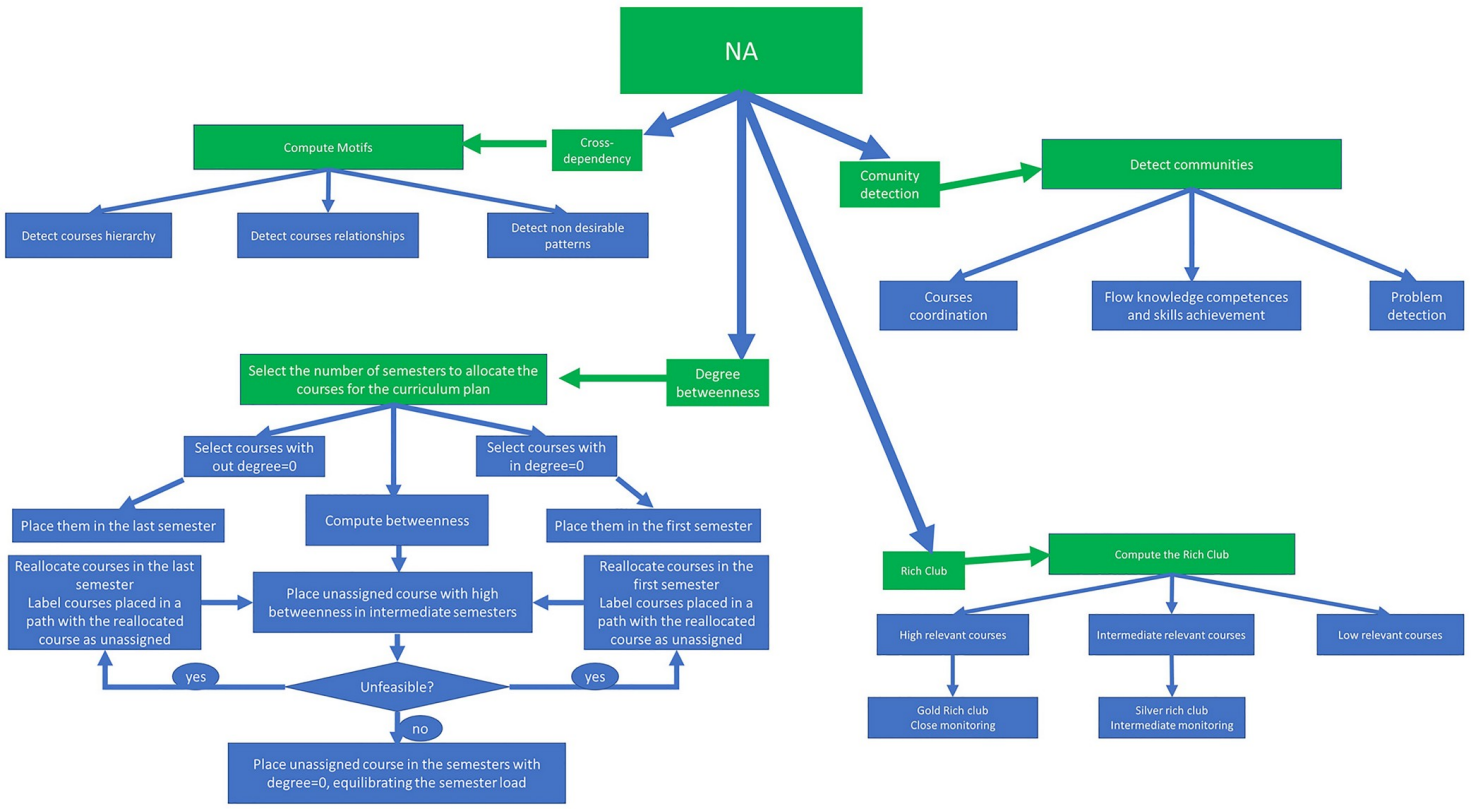
NA applications to curriculum design.

Once the information is obtained, we aggregate the expert opinion (in our real case application this group was composed by 43 professors with more than 5 years of experience) to have the final matrix relations among the courses in the curriculum plan. The second step it to apply network analysis tools for a better understanding of the complex structure of the curriculum plan. Fig 3 represents different analysis derived from different measures that can be applied to the curriculum network.

Finally, the information and the associated conclusions are presented to the experts/professors for a better understanding of the complex structure and if it is necessary (due to some inconsistencies or unfeasibility in the semesters allocations) change the content of some courses reduce these inconsistencies in an iterative way.

In order to represent the courses and the influences between different courses, throughout this paper we consider a *weighted directed network* *G* = (*X*, *E*, λ), where *X* = {1, …, *n*} is the set of *vertices* or *nodes*, *E* ⊆ *X* × *X* is the set of *arcs* or *edges* and λ is a function λ: *E* → [0, + ∞) such that for each arc (*i*, *j*) ∈ *E*, the coefficient λ(*i*, *j*) is called *weight* of (*i*, *j*). The graph is built as follows:

- Courses to be considered will be represented as nodes in the network.
- The network must reflect the relationships between courses. Since relationships between courses are directional, they are represented in the graph by directed arcs. That the contents of course *i* should precede the contents of course *j* is represented in the graph as a directed arc (*i*, *j*) from node *i* to node *j*.
- The weight of the arc λ(*i*, *j*) reflects the degree of influence/dependency between two courses.
- The set of arcs is *E* = {(*i*, *j*)|λ(*i*, *j*) > 0}.

The network must reflect the flow of knowledge as a sequence of acquisition and improvement of professional skills. Furthermore, isolated nodes in the graph can be allocated in any desirable semester in curriculum design and nodes with many relations in the structure modeled by the graph should be priorized in the allocation with respect to the rest of the nodes in the graph, since they will have more restrictions. Inconsistencies can be detected if exist a directed arc (*i*, *j*) with λ(*i*, *j*) > 0 and a directed arc (*j*, *i*) with λ(*j*, *i*) > 0. We consider the length of the path between courses *i* and *j* as the minimum number of semesters necessary to allocate the courses respecting the dependence between courses when avoiding interdependencies in a given semester.

**Fig 4:**
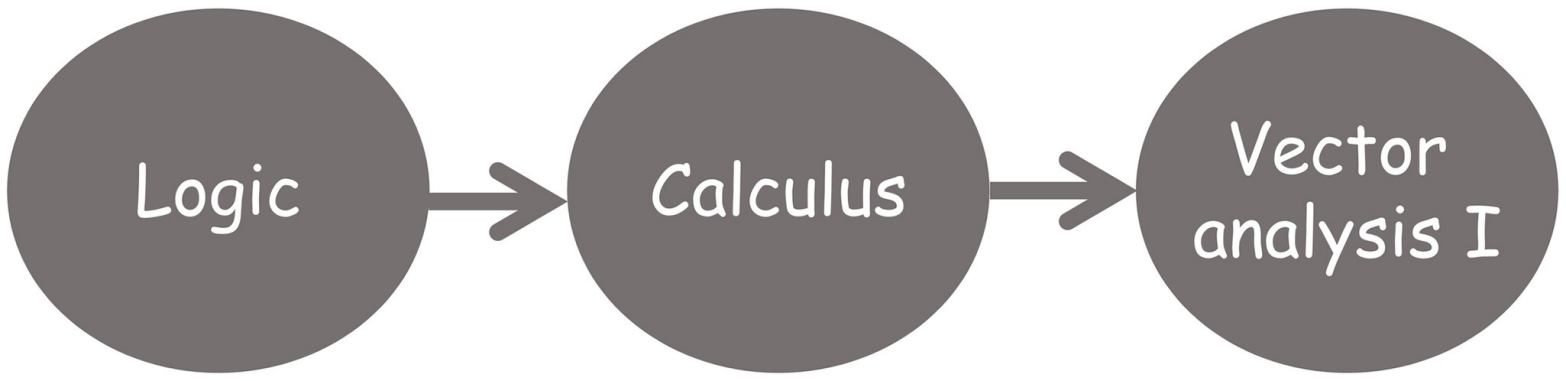
Example of assignment of intermediate courses in the study plan.

**Fig 5:**
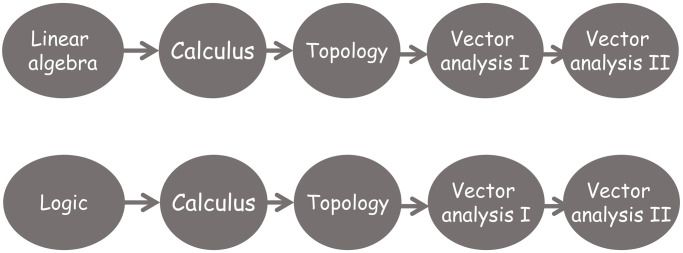
Example of allocation of course Calculus in a training itinerary taking into consideration the maximum length of the path that joints the course with any other course in the associated dependency graph.

**Fig 6:**
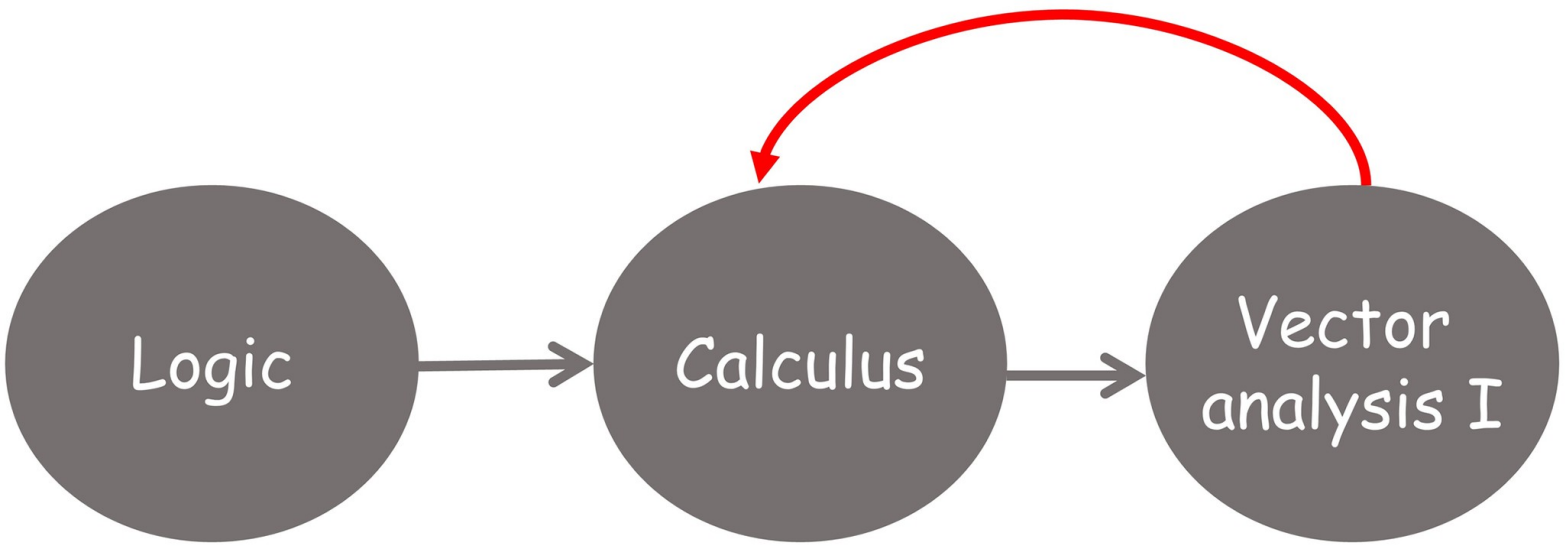
Example of non-desirable arcs.

**Fig 7:**
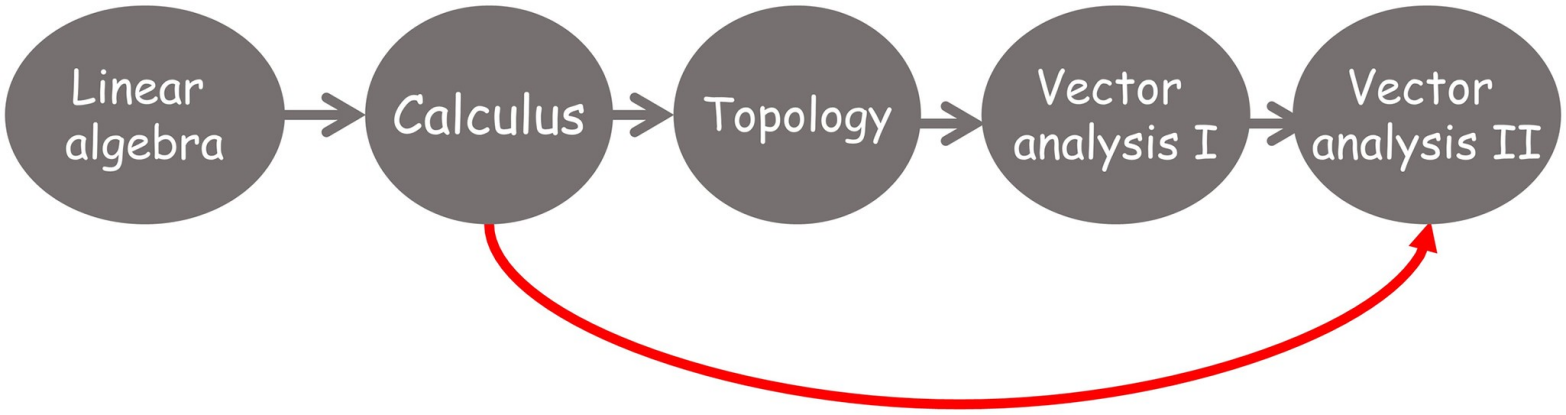
Example of non desirable assignment of courses to minimize the total length of paths joining dependent courses.

In the final phase, we can proceed to the assignment of nodes from the graph to semesters in the study plan besides other applications derived from Network Analysis. For the semester allocation problem, we impose the following restrictions (based in the Spanish Laws, which could be modify according to other countries legislation):

- The first four semesters of the degree must contain the transversal (common to all students of the same University, regardless the Degree they are studying) and basic training courses (those courses common to most degrees but adapted to the specific content of a given degree).
- If a course is considered as intermediate (i.e. there is at least one course that requires the competencies acquired by studying it) then it cannot appear in the second semester of the fourth year (see Fig 4).
- The maximum length of a path that joins a course with any other course in the dependency graph associated to the training itinerary determines the earliest semester for this course to be allocated in the degree programme (see Fig 5).
- An arc’s direction reflects the sequential acquisition of competencies so an arc cannot return to a prior semester (see Fig 6).
- It is desirable that the distance between semesters of co-requisite courses be as short as possible in the study plan (see Fig 7).

## Case study: The Mathematics Curriculum Network (MCN) in the University Rey Juan Carlos

The University Rey Juan Carlos (URJC) offered a mathematics degree for the first time in the academic year 2015-16. Previously, the URJC had offered a mathematics degree only as a part of joint degrees with computer science or software engineering. After these joint degree programs had been initiated, their coordinators carried out a series of meetings to respond to reports issued by the regional oversight authority Madrid+d (Fundación para el Conocimiento madri+d, Madrid agency for to contribute to make the quality of higher education, science, technology and innovation key elements of the competitiveness and well-being of citizens) requiring the implementation of a single degree in mathematics. To take into account student demands for modifications to the degree programs and other suggestions for changes to the existing training itineraries, several teaching coordination and curriculum design committees, constituted by qualified teachers and student representatives, met to analyze proposals. Any proposed study plan must meet certain predetermined organizational requirements. Five 6 credit courses must be assigned to each of its first six semesters. While its seventh and eighth semesters may include as elective courses any admissible course, they must also include professional internships and the final project. Additionally, the URJC imposes as a graduation requirement to pass a foreign language course known as modern language. This requirement must be met by the completion of a year-long 6 ECTS (European Credit Transfer and Accumulation System) courses during the first two years of study. Therefore, a total number of 31 courses have to be allocated in the study plan and the diameter of the final network must be less than 7 to ensure the legal requirements are satisfied.

These requirements identify 31 nodes in the **Mathematical Curriculum Network** (MCN) corresponding to the courses appearing in the mathematics degree. In order to determine the dependencies between the courses constituting the degree, personal interviews were conducted with the teachers who lectured in the degree. On the basis of these interviews, the dependency arcs were constructed and their weights were determined so as to indicate an estimated percentage of dependency of the contents of a course on the contents of previously studied courses. The weights of a given arc (i,j) represents the percentage of contents of a course i required to be fluent before a student enrols in a course j so as to succeed in course j. For an arc (*i*, *j*), for which course *i* must precede course *j* in the itinerary, three levels of dependency were established:

- Level 1: The content of course *j* requires less than 30% of the content taught in course *i*.
- Level 2: The content of course *j* requires between 30% and 75% of the content taught in course *i*.
- Level 3: The content of course *j* requires more than 75% of the content taught in course *i*.

In Table 1 we show the dependencies between the different courses in the mathematics degree in the URJC and the subscript indicates the level of percentage of matters of precedence courses necessary to understand the contents of given course.

**Table 1:** Influences between the different courses in the mathematics degree in the URJC and level of dependency.

Lecture	Relies on Material From	Supports Material In
Advance Algebra Structures (AAS)	*AS*_3_, *LA*_2_, *DM*_2_	
Algorithm Design and Analysis (ADA)	*IP*_1_, *SMMOR*_1_, *PM*_2_, *DM*_1_	
Affine Geometry (AG)	*LA*_3_	*CG*_2_, *ODE*_1_, *SS*_1_
Algebra Structures (AS)	*DM*_2_, *L*_2_	*AAS*_3_, *ODE*_2_
Biological fundamentals (BF)		
Calculus (C)	*L*_1_, *LA*_1_	*P*_2_, *T*_2_, *VAI*_3_, *CF*_2_, *PF*_2_, *ODE*_2_, *VAII*_3_, *CVFA*_3_, *MS*_2_, *SS*_3_, *PDE*_2_, *NM*_2_
Chemical Fundamentals (CF)	*C*_2_	
Complex Variables and Functional Analysis (CVFA)	*C*_3_, *VAI*_2_, *VAII*_2_, *SS*_1_, *LA*_2_, *ODE*_1_, *T*_2_	*PDE*_2_, *CG*_1_
Computational Geometry (CG)	*T*_2_, *LA*_2_, *CVFA*_1_, *AG*_2_	
Discrete Mathematics (DM)		*AS*_2_, *P*_1_, *AAS*_2_, *T*_1_, *FL*_1_, *PF*_2_, *IDM*_1_, *ADA*_1_, *SMMOR*_1_
Ethics (E)		
Formal Languages (FL)	*DM*_1_, *L*_3_, *LA*_2_	
Information and Data Modelling (IDM)	*L*_1_, *DM*_1_, *IP*_2_	*SM*_1_
Introduction to Programming (IP)	*L*_1_	*PM*_2_, *ADA*_1_, *IDM*_2_, *SM*_1_
Linear Algebra (LA)		*VAI*_2_, *CVFA*_2_, *VAII*_2_, *SS*_1_, *C*_1_, *P*_1_, *AG*_3_, *SMMOR*_1_, *T*_1_, *ODE*_2_, *MS*_1_, *PDE*_2_, *CG*_2_, *AAS*_2_, *FL*_1_, *NM*_1_
Logic (L)		*C*_2_, *AS*_2_, *IP*_1_, *FL*_1_, *IDM*_1_
Mathematical history (MH)		
Mathematical Statistics (MS)	*C*_2_, *VAI*_2_, *LA*_1_, *P*_3_	
Modern languages (ML)		
Numerical Methods (NM)	*PDE*_2_, *ODE*_2_, *VAI*_2_, *C*_2_, *LA*_1_	
Ordinary Differential Equations (ODE)	*C*_2_, *LA*_1_, *VAI*_2_, *VAII*_3_, *AS*_1_, *AG*_1_, *SS*_2_	*PDE*_3_, *NM*_2_, *CVFA*_1_
Partial Differential Equations (PDE)	*ODE*_3_, *VAI*_2_, *C*_2_, *VAII*_2_, *LA*_1_, *SS*_2_, *CVFA*_2_	*NM*_2_
Physics Fundamentals (PF)	*C*_2_, *DM*_2_	
Probability (P)	*C*_2_, *DM*_1_, *LA*_1_	*MS*_3_
Programming Methodology (PM)	*IP*_2_	*ADA*_1_, *SM*_2_
Software Modelling (SM)	*IP*_1_, *PM*_2_, *IDM*_1_	
Statistical Methods for Management and Operations Research (SMMOR)	*DM*_1_, *LA*_1_	*ADA*_1_
Surfaces and Shapes (SS)	*C*_3_, *VAI*_3_, *AG*_1_, *T*_3_, *LA*_1_	*ODE*_2_, *CVFA*_1_, *PDE*_2_
Topology (T)	*C*_2_, *LA*_1_, *DM*_1_	*VAI*_1_, *CVFA*_2_, *CG*_2_, *VAII*_2_, *SS*_2_
Vector Analysis I (VAI)	*C*_3_, *T*_1_, *LA*_2_	*VAII*_3_, *ODE*_3_, *PDE*_2_, *CVFA*_2_, *SS*_3_, *MS*_2_, *NM*_2_
Vector Analysis II (VAII)	*VAI*_3_, *C*_3_, *LA*_2_, *T*_1_	*ODE*_3_, *CVFA*_2_, *PDE*_2_

The transversal and basic training courses are LA, IP, C, PF, E, BF, MH, AVI, CF and ML.

**Fig 8:**
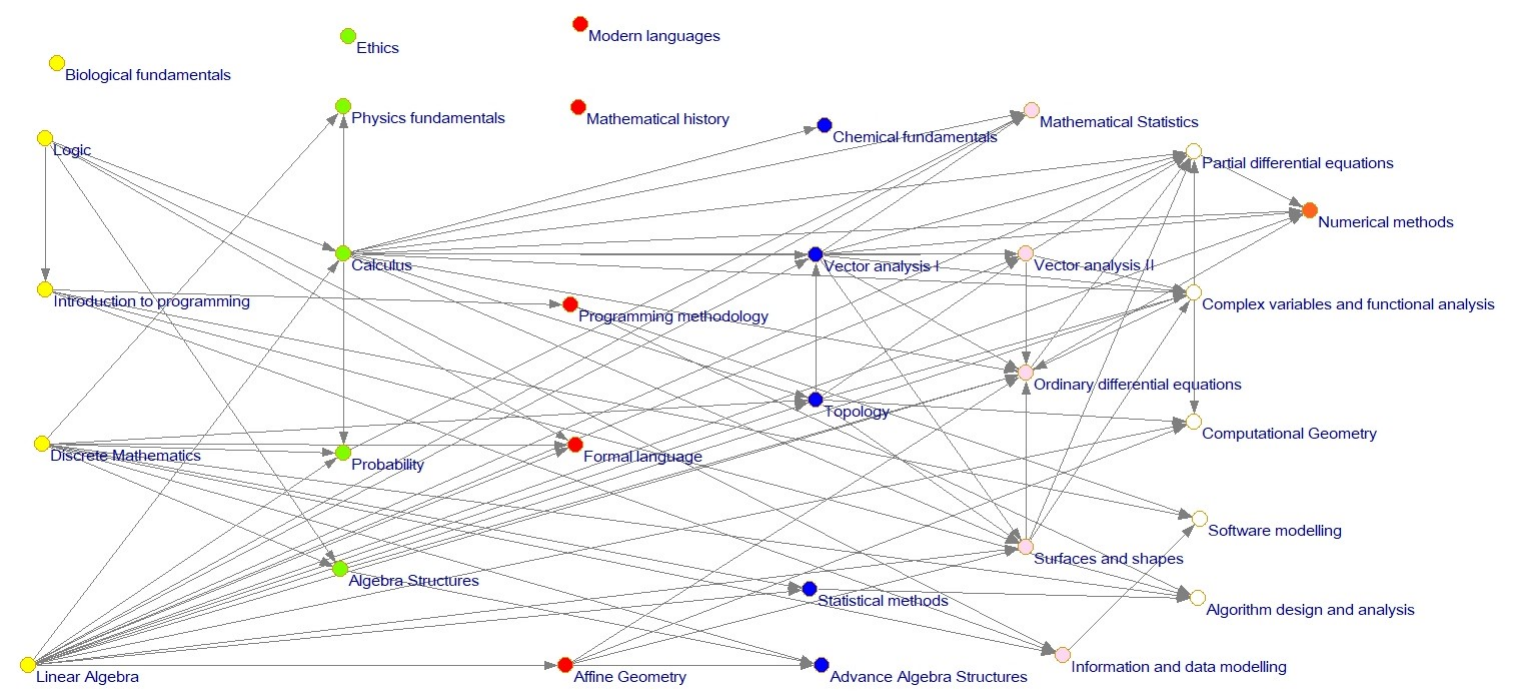
Level 1, 2 and 3 math curriculum network.

Once this information was obtained, the curriculum network was built (see Fig 8). Each semester is plotted in a different color for clearness. Table 2 presents the color description for each semester.

**Table 2:** Color description for each course in the math curriculum network.

Color	Semester
Yellow	First semester
Green	Second semester
Red	Third semester
Blue	Fourth semester
Pink	Fifth semester
White	Sixth semester
Orange	Seventh semester

**Fig 9:**
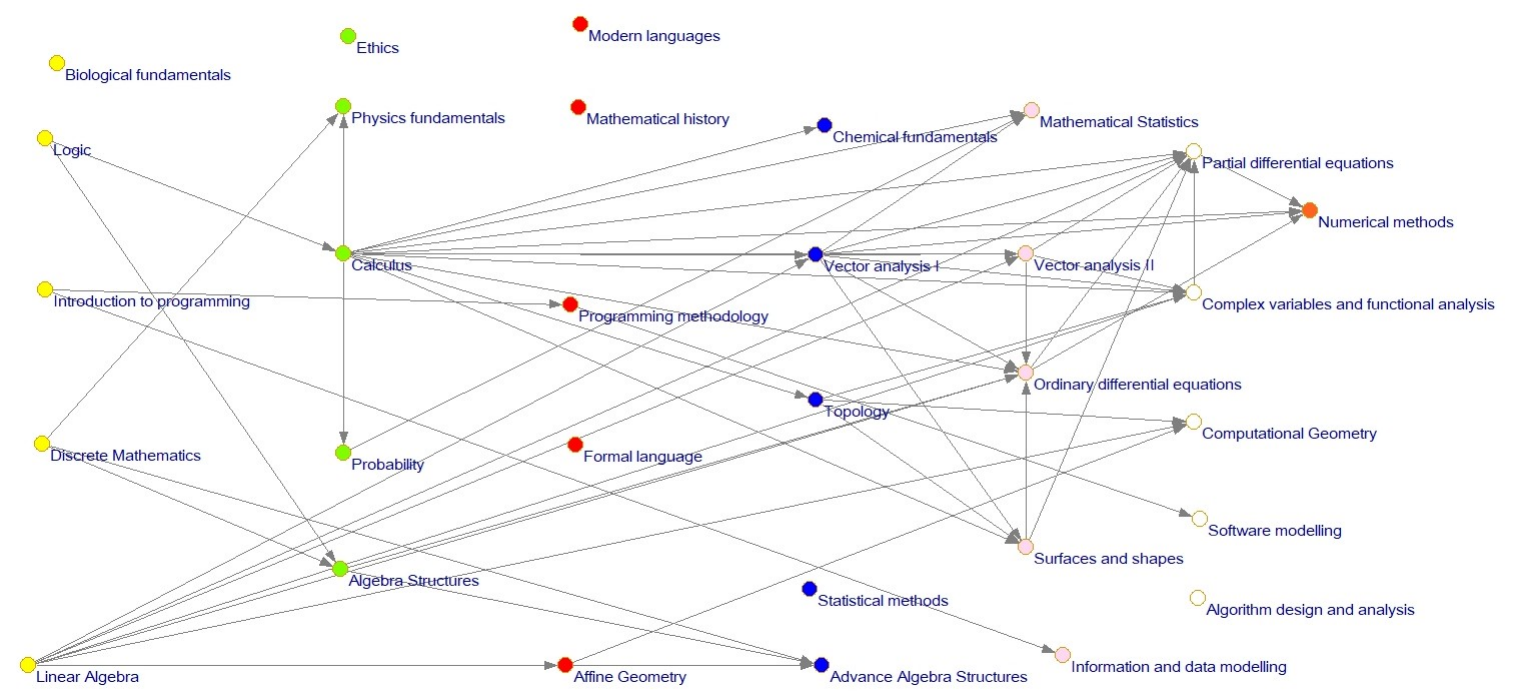
Curriculum network level 2 and 3 influence relation.

**Fig 10:**
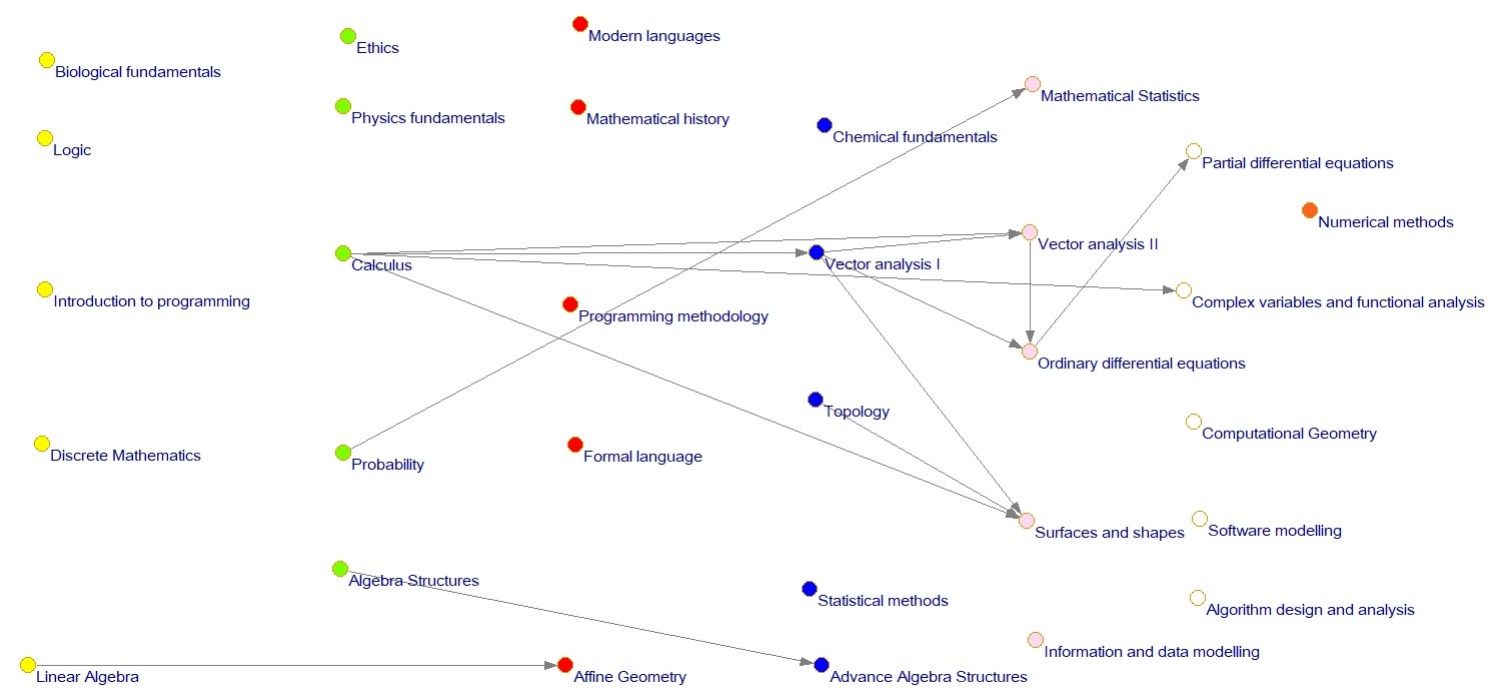
Curriculum network level 3 influence relation.

The network evaluation allows visualization of the entire network and also its subnetworks in which there are considered only dependencies between courses of level 1 or several levels (see Figs 9 and 10).

## Results

Once the dependency structure of the URJC mathematics degree has been modeled by means of a network (that we have called MCN), in this section we present a network analysis to deal with the objectives pointed out in the introduction. We are going to divide this section into 4 differentiated subsections according to the different analyzes presented that are: centrality measures analysis of the MCN, community detection analysis of the MCN, mofits indentification and global network analysis.

### Centrality measures analysis of the MCN

First at all, let us note the Mathematical Curriculum Network (MCN) is a directed and weighted network. Also let us note that the direction of the link represent some dominance status, since the link between courses i and j exist if and only if there it is necessary to pass the knowledge of course i first to be able to understand or to pass the course j. As a consequence of this fact, some classical centrality measures based on minimal paths as: closenesss or betweeness are not appropriate for this particular case. Taking all this in consideration, we are going to measure the importance of the courses in this network based on two classical centrality measures that deals with directed and weighted networks: the degree and PageRank centrality measure. In directed networks, in-degree and out-degree are extremely local measures, although quite informative. In this sense, intermediate measures as flow betweenness can be understood as a more general and robust measure than in degree.

In Table 3, we can see the importance/relevance of each course in the Mathematical Curriculum Network in terms of degree and PageRank value.

**Table 3:** Main centrality measures for the study plan of the URJC mathematics degree.

Course	Total degree	Out-degree	PageRank	Flow betwenness
Linear Algebra	16	16	0.124	0
Calculus	14	12	0.104	2.5
Vector Analysis I	10	7	0.042	1
Ordinary differential equations	10	2	0.021	0
Discrete Mathematics	9	9	0.068	1
Complex Variables and Functional Analysis	9	3	0.021	0
Topology	8	5	0.035	1
Surfaces and Shapes	8	3	0.021	0.5
Partial differential equations	8	1	0.018	0
Vector Analysis II	7	3	0.024	0
Logic	5	5	0.137	0
Introduction to Programming	5	4	0.052	0
Numerical Methods	5	0	0.015	0
Affine Geometry	4	3	0.022	0
Algebra Structures	4	2	0.023	1.5
Probability	4	1	0.021	0
Information and Data Modelling	4	1	0.019	1
Mathematical Statistics	4	0	0.015	0
Computational Geometry	4	0	0.015	0
Algorithm Design and Analysis	4	0	0.015	0
Programming Methodology	3	2	0.025	0
Statistical Methods	3	1	0.018	1
Advance Algebra Structures	3	0	0.015	0
Formal Language	3	0	0.015	0
Software Modelling	3	0	0.015	0
Physics Fundamentals	2	0	0.015	0
Chemical Fundamentals	1	0	0.015	0
Biological Fundamentals	0	0	0.015	0
Ethics	0	0	0.015	0
Mathematical History	0	0	0.015	0
Modern Languages	0	0	0.015	0

The results highlight the importance of the classes Linear Algebra, Calculus, Vector Analysis I, Ordinary Differential Equations and Complex Variable, and Vector Analysis. Student performance in these courses must be monitored closely since a large number of future courses depend on them. To detect the core courses of the curriculum, the flow betweenness measure on the original network was calculated for minimum paths. This emphasizes the importance of the courses Calculus, Algebraic Structures, Vector Analysis I, Discrete Mathematics, Topology, Data and Information Modelling, Statistical Methods, and Curves and Surfaces as these courses appear in the minimum dependency path between any pair of courses in the degree. To detect the instrumental and terminal courses of the curriculum, the degree-out measure on the original graph was calculated. This highlights the importance of the courses Linear Algebra, Calculus, and Discrete Mathematics as “key” courses in the degree, which should be positioned at the beginning of the curriculum, preferably in the first semester of study. Those courses that achieve a score of zero can be considered terminal courses in the curriculum and can be placed in the final semesters of the curriculum.

### Community detection analysis of the MCN

Community detection in NA are beneficial for numerous applications such as finding common characteristics between nodes or finding sets of nodes with similar interactions.

In order to determine the course groups according to the dependencies between the syllabus of the courses in the curriculum, a community analysis using the Louvain algorithm [39] was made. The community detection problem in direct network has been studied in less depth than in the non-directed case. For this reason we have performed a naive transformation (removing the direction) to be able to apply the classic Louvain algorithm defined for non-directed networks. Removing the directionality of the edges to identify communities in a directed network is one of the most common ways to identify communities that empower those groups with many relationships between their members and few with members from abroad. We have simulated 10000 random graphs generated with the same number of vertices and links to determine the degree of modularity of our network using the Erdös-Rènyi model [40]. Other algorithms to generate random graphs such as configurational [41] or Chung-Lu [42] can be used, were the connectivity is specify by the degree sequence $\vec{k}$ and many patterns vary with the mean (*k*) and variance *k*^2^) of this sequence. In this work, we have adopted the model proposed by Erdös-Rènyi for simplicity. Table 4 presents the main descriptive statistics of 3 different community detection algorithms modularity in the random networks, where Betweenness refers to the algorithm propose in [43], external optimization ([44–46]) and Lovain ([39]) community detection algorithms.

**Table 4:** Descriptive statistics of Community detection algorithms modularity applied to 10000 random graphs with the same vertices and links as the curriculum network (null model).

Community detection Algorithm	Average	Standard Deviation	Average Confidence interval (*IC*95%(*μ*))
Betweenness	0.261	0.039	[0.2601019; 0.2616425]
External optimization	0.124	0.052	[0.1237327; 0.1258032]
Louvain	0.243	0.105	[0.2410161; 0.2451377]

The degree of modularity is moderate (0.32), which reflects the fact that we have a modular network with clearly identifiable communities, even though there are relations between the communities, which means that there are also dependencies between the communities.

**Fig 11:**
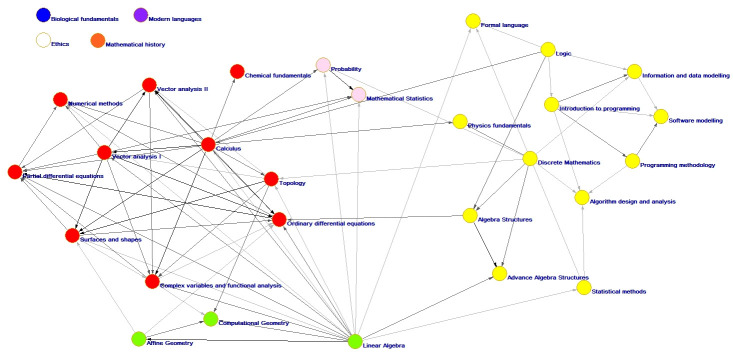
Community network describing the curriculum of the URJC mathematics degree.

In Fig 11, we present a visualization of the optimal solution presented by the Louvain algorithm.

It is worth noting the presence of four groups of courses (see Fig 11), which could be named as follows:

- Group 1: Basic Algebra
- Group 2: Probability and mathematical statistics
- Group 3: Advanced computing and algebra
- Group 4: Vector calculus and numerical methods

The courses Biological Foundations, Ethics, Modern Language, and History of Mathematics are separated from the other courses in the degree.

### Global network analysis

**Fig 12:**
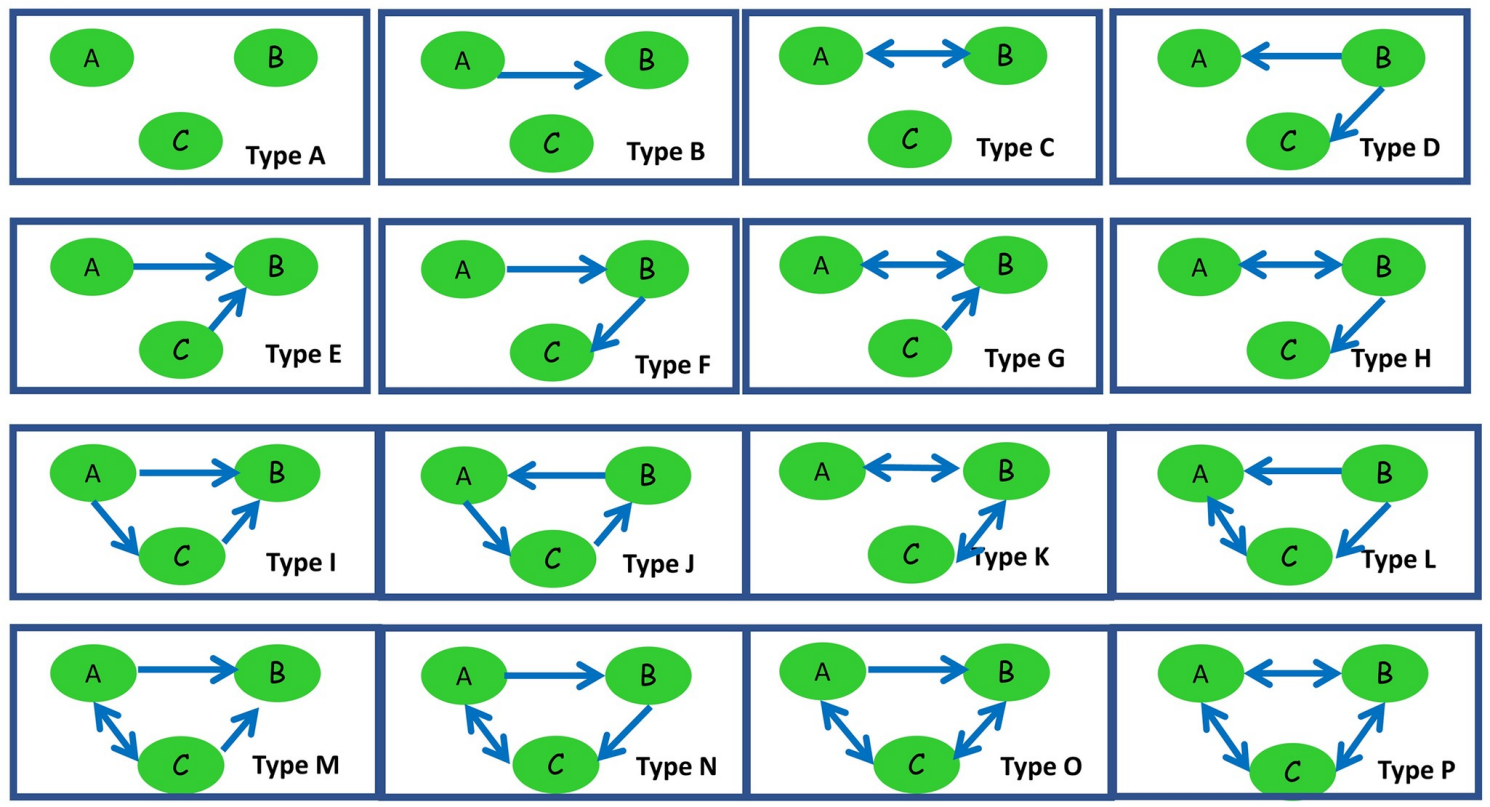
Value chart of Φ(*k*) according to *k*.

The rich club in the network, represented by the principal courses in the study plan, refers to the tendency of the dominant elements of the system to form tightly interconnected groups. The maximum value for Φ(*k*) with minimum *k* value is attained for *k* = 10 where *R*(10) is the set of courses Linear Algebra, Calculus, Vector Analysis I, Ordinary Differential Equations (see Fig 12).

The assortativity measure of the net is 0.12 (p-value 0.228, associated to the null hypothesis: assortativity = 0 vs the alternative assortativity ≠0). Assortative measure is defined here as the correlation coefficient between the degree of adjacent nodes. As the assortative measure is low, we can conclude there is a disassortative relationship between the nodes of the net, since nodes with high degree tend to connect with low degree nodes.

### Cross-dependencies analysis

Some networks require a predefined model to determine the influence of one region on another due to temporal dependencies. Cross-dependencies helps to identify the network’s hierarchy of influence.

**Fig 13:**
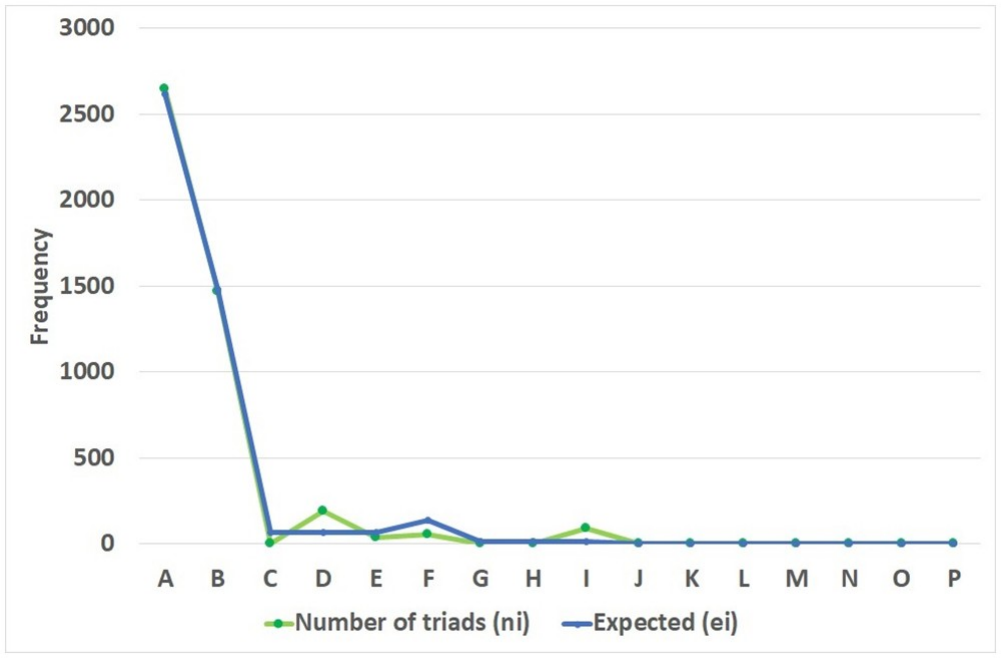
Types of motifs of size three in a random graph.

**Fig 14:**
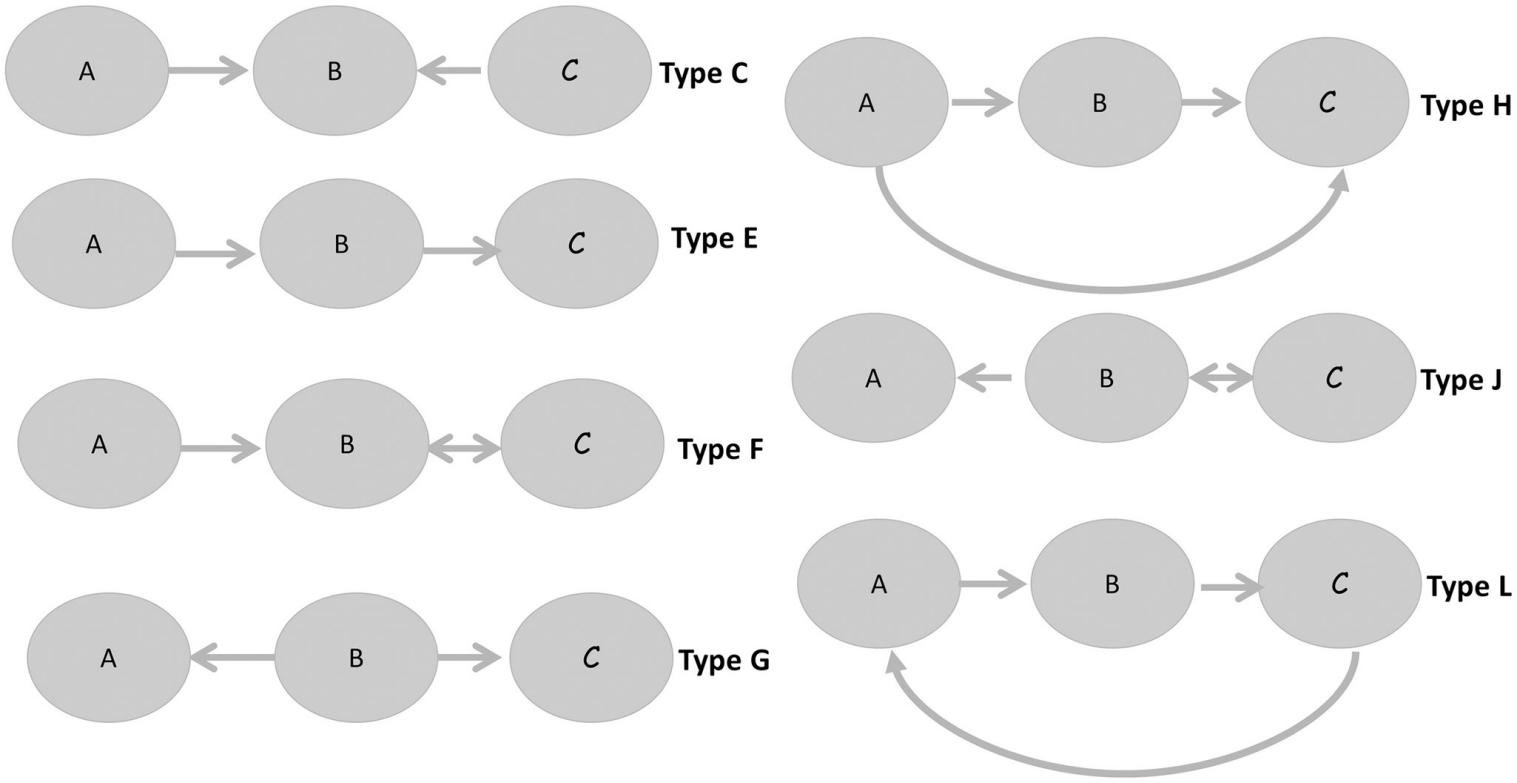
Number of average motifs and expected with three vertices in a random graph compared to the study plan graph.

To detect cross-dependencies between classes, motifs of size three were considered. We compared the number of expected number of motifs of type 3 in a random graph with the observed number of motifs of size 3 in the study plan graph (see Figs 13 and 14). The random model used has been generated using the same number of nodes and arcs found in the original network using Pajek 5.1 software. Triad census are labelled according to [38].

**Fig 15:**
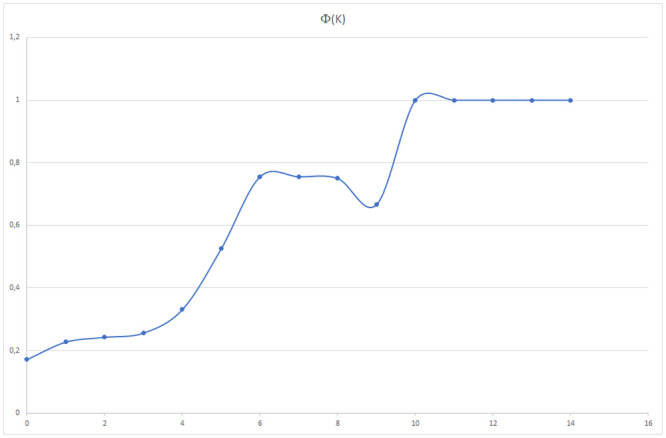
Motifs differing significantly between a random graph and the study plan graph.

Significant differences are apparent for patterns D, E, F and I (p-value < 2*E* − 16) (see Fig 15). Patterns E and F were significantly smaller respect to a random graph in the study plan. It is desirable to avoid patterns C, G, H, K, L, M, N, O and P in the study plan as these patterns exhibit significant course interdependency, obliging considerable coordination between professors, something which is not always easy to achieve. Similarly, pattern type J should be avoided because of its cyclic structure, which would obligate use of knowledge taught in courses to be situated in semesters posterior to that in which it would be needed. Significant differences in patterns D and I were found to be higher in the study plan graph than in random graphs. This is a desirable property, as it reflects that knowledge will flow correctly provided that the corresponding courses are assigned to different semesters.

## Conclusion

In this paper we have proposed a new tool to visualize a curricula design from a network analysis point of view, by using the natural tools and concepts from Graph Theory. Even if just some studies can be found that combine network analysis with tools for curricula design, the methodology proposed here provides a new vision of the structure and functionality of different curriculum designs. In particular, we propose to construct the graph in a first step following the recommendations given in section 3, check for inconsistencies helped by the graph visualization, and if the study plan satisfies all the requirements proceed with network analysis to enrich the information of performance, otherwise return to the previous stage to reallocate courses in the semesters until all the requirements are satisfied.

From this network analysis, it is possible to detect incongruences or mistakes in the study plan in an automatic way. From a node network analysis point of view we can identify or detect the main courses in the study plan or the courses that required a detailed performance monitoring since the influence in other courses is high. Also it is important to mention that the relation between nodes in this network is based on the necessities of one course t respect the others. This community detection algorithm allows professors to coordinate in the case they are involved teaching the same subjects. In this sense the natural groups of courses could be identified in a natural way after a community detection procedure in the courses network of a study plan.

Finally it also relevant to mention that the general or topological properties of the whole network provide an interesting information of the whole study plan. Topological network measures as the density of the network, assortativity degree among others permits to know for example if the relations between courses in the study plan are more probable among similar courses or the opposite.

One limitation of the present study is the consideration in the model mandatory courses antecedence requisites. We find this issue as a very interesting task in a future research line.

This article has provided a step-by-step procedure for analyze the key courses in a study plan (deciding if it is recommendable small modifications or not), identify the natural groups of courses that should be coordinate, detects incongruences in the plan, robustness of the plan study or the level of connections of the plan allowing the comparison from an structure organization point of view different plans to decide what we want to design. Still, the problem of meeting all the requirements could be non-trivial and a future research item is to extend this work with a detailed algorithm to assign the courses to semesters efficiently.

## Supporting information

S1 Data (XLSX)
